# Going with the flow: New insights regarding flow induced K^+^ secretion in the distal nephron

**DOI:** 10.14814/phy2.70087

**Published:** 2024-10-20

**Authors:** Samia Lasaad, Andrew J. Nickerson, Gilles Crambert, Lisa M. Satlin, Thomas R. Kleyman

**Affiliations:** ^1^ Department of Pediatrics Icahn School of Medicine at Mount Sinai New York New York USA; ^2^ Department of Medicine University of Pittsburgh Pittsburgh Pennsylvania USA; ^3^ Centre de Recherche Des Cordeliers, Institut National de la Santé et de la Recherche Scientifique (INSERM) Sorbonne Université, Université Paris Cité, Laboratoire de Physiologie Rénale et Tubulopathies Paris France; ^4^ Unité Métabolisme et Physiologie Rénale Centre National de la Recherche Scientifique (CNRS) EMR 8228 Paris France; ^5^ Department of Cell Biology and Department of Pharmacology and Chemical Biology University of Pittsburgh Pittsburgh Pennsylvania USA

**Keywords:** BK channels, Calcium signaling, Mechanotransduction, PIEZO1, TRPV4

## Abstract

K^+^ secretion in the distal nephron has a critical role in K^+^ homeostasis and is the primary route by which K^+^ is lost from the body. Renal K^+^ secretion is enhanced by increases in dietary K^+^ intake and by increases in tubular flow rate in the distal nephron. This review addresses new and important insights regarding the mechanisms underlying flow‐induced K^+^ secretion (FIKS). While basal K^+^ secretion in the distal nephron is mediated by renal outer medullary K^+^ (ROMK) channels in principal cells (PCs), FIKS is mediated by large conductance, Ca^2+^/stretch activated K^+^ (BK) channels in intercalated cells (ICs), a distinct cell type. BK channel activation requires an increase in intracellular Ca^2+^ concentration ([Ca^2+^]_i_), and both PCs and ICs exhibit increases in [Ca^2+^]_i_ in response to increases in tubular fluid flow rate, associated with an increase in tubular diameter. PIEZO1, a mechanosensitive, nonselective cation channel, is expressed in the basolateral membranes of PCs and ICs, where it functions as a mechanosensor. The loss of flow‐induced [Ca^2+^]_i_ transients in ICs and BK channel‐mediated FIKS in microperfused collecting ducts isolated from mice with IC‐specific deletion of *Piezo1* in the CCD underscores the importance of PIEZO1 in the renal regulation of K^+^ transport.

## 1 INTRODUCTION

**FIGURE 1:**
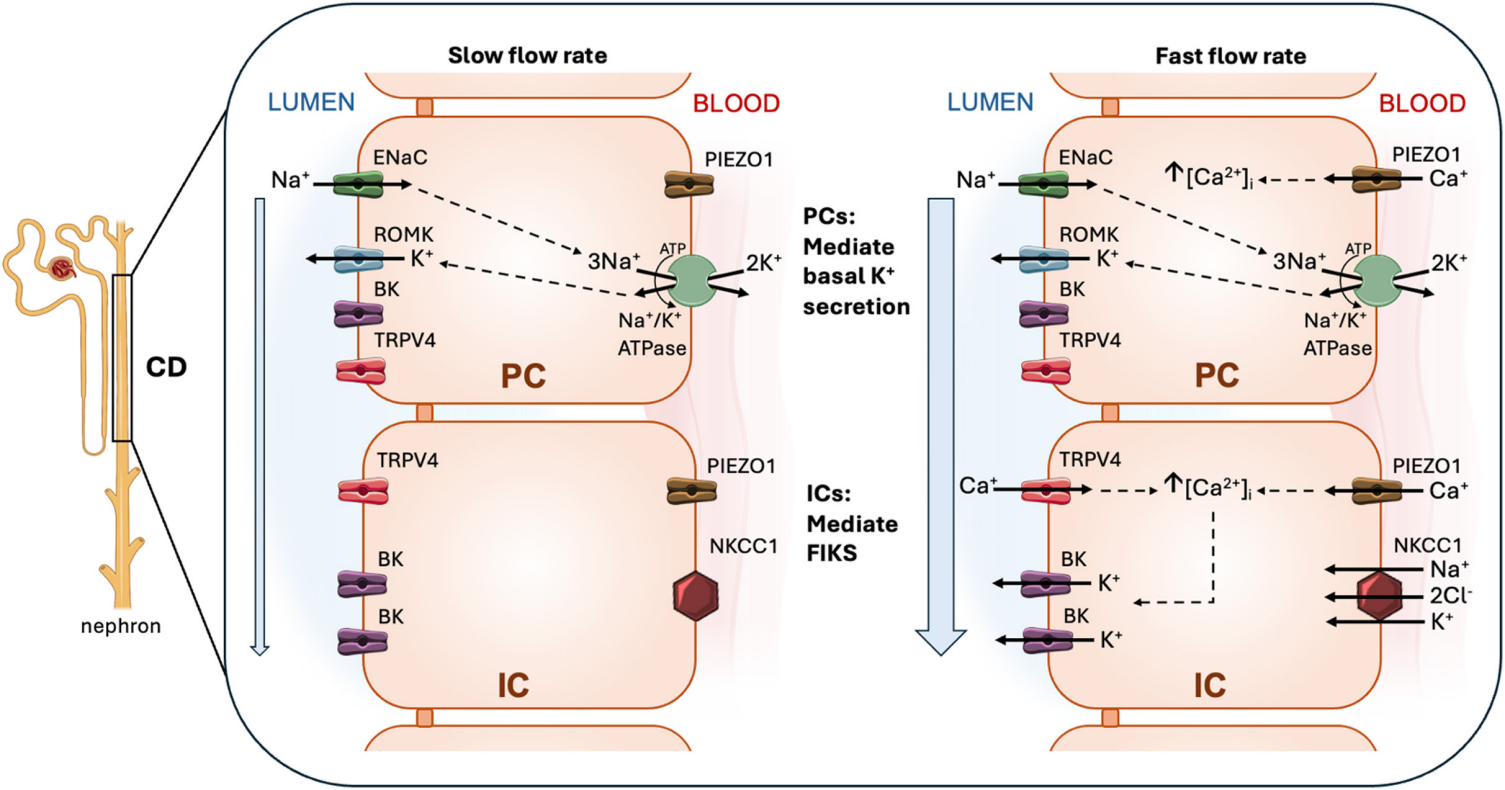
Sites of transport proteins mediating basal and flow induced K^+^ secretion (FIKS) in the mammalian collecting duct (CD). In the CD, under slow flow conditions (left), principal cells (PCs) reabsorb Na^+^ through apical ENaC channels. Na^+^ exits the cell at the basolateral membrane, in exchange for K^+^ uptake, via the basolateral Na^+^,K^+^‐ATPase. A favorable electrochemical gradient promotes apical K^+^ secretion through apical ROMK channels. Under fast flow conditions (right), intracellular Ca^2+^ concentration ([Ca^2+^]_i_) increases in intercalated cells (ICs) due to mechanoactivation of basolateral PIEZO1 and opening of TRPV4 channels, leading to external entry of Ca^2+^, as well as internal store release of Ca^2+^. Elevated [Ca^2+^]_i_ activates FIKS through apical BK channels.

The kidney is the major organ responsible for sustaining total body K^+^ homeostasis, critical for maintaining normal blood pressure and neuromuscular excitability. In adults, the kidneys generally achieve a state of “net zero” K^+^ balance by matching urinary K^+^ excretion with K^+^ intake (Giebisch, 1998). Approximately 80%–90% of filtered K^+^ is passively reabsorbed along the proximal tubule and thick ascending loop of Henle (TAL). The remaining K^+^ in the tubular fluid ultimately reaches the late distal convoluted tubule (DCT2), connecting tubule (CNT), and cortical collecting duct (CCD), traditionally referred to as the aldosterone‐sensitive distal nephron (ASDN). Recent evidence suggests that the DCT2 and early CNT may compose the aldosterone‐insensitive distal nephron (AIDN), potentially regulated by glucocorticoids (reviewed in (Demko et al., 2024)). However, the distinction between ASDN and AIDN segments may be dynamic and could depend on physiologic conditions, such as K^+^/Na^+^ intake, which can affect the plasticity and extent of the ASDN. For the purpose of this review, we will refer to these segments as the traditional ASDN. These latter portions of the nephron are responsible for the final coordinated renal regulation of K^+^ and Na^+^ (Lasaad & Crambert, 2023). Here, K^+^ can either be secreted or reabsorbed, depending on physiological needs. Indeed, the filtered load of K^+^ is almost completely reabsorbed by the proximal tubule and the TAL. A mere 10% of the filtered load of K^+^ leaves the TAL and arrives in the ASDN. Under classical conditions, in the human with a normal GFR and plasma K^+^ value, it is estimated that approximately 70 mmol of K^+^ arrives to the distal nephron, a value very close to the mean dietary intake. Therefore, to remain in K^+^ balance in the face of excess intake, secretory pathways are activated in the distal nephron (Figure 1) (Palmer, 2015).

Baseline K^+^ secretion into the tubular fluid relies on two processes localized to principal cells (PCs) in the ASDN: (1) the generation of a favorable electrochemical K^+^ gradient, facilitated by electrogenic Na^+^ transport via the apical epithelial Na^+^ channel (ENaC) and basolateral Na^+^/K^+^‐ATPase and (2) an apical membrane permeability to K^+^ (Johnston et al., 2023; Yang et al., 2021). Electrophysiologic studies of the CNT/CCD have identified two apical K^+^‐selective channels: a low‐conductance, constitutively active renal outer medullary K^+^ (ROMK) channel (Frindt & Palmer, 1989; Wang et al., 1990), and a large conductance, Ca^2+^/stretch‐activated K^+^ (BK) channel, also known as the maxi‐K channel (Hunter et al., 1984; Pacha et al., 1991). Although expressed within the same nephron segment, these channels fulfill distinct physiological roles in distinct cell types in maintaining K^+^ balance.

## 2 PARALLEL K^+^ SECRETORY PATHWAYS IN THE DISTAL NEPHRON

ROMK channels, localized in Na^+^‐absorbing PCs, are responsible for constitutive baseline K^+^ secretion (Welling, 2013). K^+^ efflux through apical ROMK channels is driven by electrogenic Na^+^ absorption via apical ENaC and the basolateral Na^+^/K^+^‐ATPase in PCs (Gray et al., 2005). K^+^ secretion in the CNT/CCD is critically dependent on Na^+^ delivery to and ENaC‐mediated absorption in this segment (Figure 1). Physiological variations in blood [K^+^] regulate activity of the Na^+^‐Cl^−^ cotransporter (NCC) in the DCT (the “potassium switch”) (Hoorn et al., 2020; Terker et al., 2015; Welling et al., 2024) and govern the rate of Na^+^ delivery to the distal nephron segments that express ENaC (i.e., DCT2, CNT, and CCD), which in turn modulates both Na^+^ absorption and K^+^ secretion (Good et al., 1984). The underlying mechanism involves WNK/SPAK‐ and protein phosphatase‐dependent regulation of NCC activity in the DCT. Increases or decreases in blood [K^+^] inhibit or stimulate NCC activity, respectively, which in turn regulates distal Na^+^ and fluid delivery. By shifting delivery of Na^+^ transport toward or away from the ENaC‐expressing segments, K^+^ excretion that is coupled to Na^+^ reabsorption can be modulated in a very rapid and reversible manner. Whole body K^+^ homeostasis is therefore achieved primarily by titrating ENaC/ROMK activity up or down to accommodate normal variations in K^+^ intake.

Recent evidence has highlighted the importance of a second apical K^+^ secretory pathway which contributes significantly to distal nephron K^+^ handling under conditions of high tubular fluid flow rates. This process, termed flow‐induced K^+^ secretion (FIKS), is mediated by large conductance, Ca^2+^/stretch‐activated BK channels located in the apical membranes of intercalated cells (ICs) in the ASDN (Figure 1) (Carrisoza‐Gaytan et al., 2020). IC BK channels are activated in response to a flow‐induced elevation in the intracellular Ca^2+^ concentration [Ca^2+^]_i_, (described in more detail below). In contrast to the constitutively active ROMK channel, with a high basal open probability (*P*_*O*_) (Welling & Ho, 2009), BK channels in ICs, closed under baseline conditions (Palmer & Frindt, 2007), are activated by an extrinsic stimulus (e.g., fluid shear stress and/or membrane stretch) as may accompany dietary K^+^ loading, acute volume expansion, and diuretic use (Carrisoza‐Gaytan et al., 2014; Liu et al., 2003). BK channel activation in response to an increase in luminal flow rate and an associated increase in [Ca^2+^]_i_ signaling leads to a large K^+^ secretory flux. While previous studies implicated BK channels in this response (Pluznick et al., 2003; Rieg et al., 2007), Carrisoza‐Gaytan et al. demonstrated that IC‐localized BK channels in the CCD are indispensable for FIKS (Carrisoza‐Gaytan et al., 2020). Discovery of IC‐mediated FIKS uncovered a new role of ICs, which were once thought to participate only in acid/base regulation.

Although the phenomenon of FIKS has been established for some time, the physiological role of this pathway, as well as many of its underlying mechanistic details, are still being uncovered through powerful in vivo and ex vivo methods applied to novel mouse models. This review focuses on recent key studies which have significantly advanced our understanding of BK channel mediated K^+^ secretion, or FIKS.

## 3 CHARACTERISTICS OF RENAL BK CHANNELS

BK channels are ubiquitous plasma membrane K^+^ channels gated by stimuli such as membrane stretch, membrane potential, and [Ca^2+^]_i_ (Frindt & Palmer, 1987; Hirsch et al., 1993; Latorre et al., 1989; Taniguchi & Imai, 1998). The functional channel is formed by a homomeric assembly of four α subunits (encoded by the *Kcnma1* gene), each consisting of a short extracellular N‐terminus, seven transmembrane segments including the voltage‐sensing (S1–S4) and pore‐forming (S5–S6) segments, and a large intracellular C‐terminus containing two RCK (regulator of K^+^ conductance) domains (Contreras et al., 2013). BKα subunits can undergo extensive alternative splicing (Latorre et al., 2017; Shipston & Tian, 2016; Whelan et al., 2024), generating a variety of isoforms with differing sensitivity to regulatory elements, including [Ca^2+^]_i_ (Erxleben et al., 2002; Soom et al., 2008), phosphorylation (Tian et al., 2001), and cys‐palmitoylation (Zhou et al., 2012). BKα subunits may also co‐assemble with or without accessory β and/or γ subunits as part of the channel complex (Gonzalez‐Perez & Lingle, 2019). BKβ1–4 (*KCNMB1‐4*) and BKγ1–4 (also referred to as *LRRC26*, *52*, *55*, and *38*, respectively) exhibit highly diverse and tissue‐specific expression patterns, each possessing a unique signature of channel modulating properties. These include effects on gating, trafficking, sensitivity to Ca^2+^ or sensitivity to the scorpion venom‐derived iberiotoxin and charybdotoxin (Lippiat et al., 2003). The many possible arrangements of alternatively spliced BKα subunits and their assembly with one or more regulatory β/γ subunits may lead to a huge functional diversity among different cell types and physiologic conditions.

Immunodetectable BK channels are expressed throughout the kidney, including PCs and ICs of the ASDN (Li et al., 2016). Pore‐forming BKα subunits have been shown to co‐localize with β1 in the CNT (Grimm et al., 2007; Pluznick et al., 2005) and with β4 subunits in ICs, as well as in the TAL and DCT (Grimm et al., 2007). BKβ1 also appears to co‐localize with BKα in the cilia of PCs of rabbit CCD (Carrisoza‐Gaytan et al., 2017). BKβ1^−/−^ mice develop hypertension, which is exacerbated by feeding a diet rich in K^+^ (Grimm et al., 2009). This effect has been attributed to an impaired ability to secrete K^+^, leading to elevated aldosterone levels and increased blood pressure. These mice also display reduced urinary K^+^ excretion in response to acute volume expansion (Pluznick et al., 2005). BKβ4 was shown to be critical for apical trafficking of BK channels in ICs in response to K^+^ loading with an alkali (i.e., KHCO_3_) diet (Wen et al., 2013). Accordingly, BKβ4^−/−^ mice display impaired urinary K^+^ excretion during K^+^ loading (Cornelius et al., 2012). Whether BKγ subunits are incorporated into PC‐ or IC‐localized BK channels is currently unknown. However, BKγ1 (Lrrc26) was shown to be critical for BK channel‐mediated K^+^ secretion in mouse distal colon, another aldosterone‐sensitive, K^+^‐excreting tissue (Gonzalez‐Perez et al., 2021).

Numerous BK splice variants have been identified in mice. One of the important coding regions where alternative splicing occurs spans exons 20–24 within *Kcnma1*. Alternatively spliced channels may either lack exons 21–23 (referred to as the “ZERO” variant), or contain exon 21 (“e21”), exon 22 (stress‐related exon “STREX” variant), exon 23 (e23), or exons 21 and 22 together (Chen et al., 2005; Xie & McCobb, 1998). Recent studies revealed that ASDN‐localized BK channels primarily consist of the ZERO and e21 α subunit splice variants (Whelan et al., 2024), although both STREX and e23 isoforms were also detected in low levels. Alternative splicing of kidney‐localized BKα transcripts at other known sites (e.g., exon 2, 10, or 18) was not apparent in these studies. ZERO and STREX isoforms of the channel are differentially affected by phosphorylation and cys‐palmitoylation events (Jeffries et al., 2010; Zhou et al., 2012). However, little is known about the relative importance of the different α subunit isoforms with respect to K^+^ secretion in the kidney. Studies using whole kidney and microdissected tubule segments (DCT2/CNT/CCD) revealed that dietary K^+^ loading upregulates each of the five splice variants (ZERO, STREX, e21, e23, and e22/23) to differing extents compared to a control diet (Whelan et al., 2024). There are other sites of alternative splicing which are known to affect channel behavior, including variations in the C‐terminal amino acid sequence. Three distinct C‐terminal variants were identified in the ASDN, ending with the amino acid sequences “VYR,” “ERL,” or “DEC.” Interestingly, the C‐terminal “DEC” variant, which exhibits a dominant negative‐like phenotype in vitro, was upregulated in the distal tubule during K^+^ loading (Whelan et al., 2024). The physiologic implications of these splicing events, which are apparently regulated by dietary K^+^, remain undetermined.

Phosphorylation plays a crucial role in modulating BK channels, as it allows a fast response to environmental changes such as acute changes in tubular flow. The BK⍺ subunit is activated by the cGMP‐dependent protein kinase PKG (Stockand & Sansom, 1996), whereas PKA, PKC, and c‐Src can either activate or inhibit the channel depending on the cell type (Bai et al., 2020; Barman et al., 2004; Hagen et al., 2003; Ling et al., 2000; Shipston et al., 1996; Shipston & Armstrong, 1996). For instance, under low‐flow conditions, BK channels are inhibited by PKA in PCs, but not in ICs (Liu et al., 2009). The difference observed may be explained by the ⍺‐splice variants and β‐isoforms expressed in these distinct cell types. Additionally, Li et al. demonstrated that the inhibition of ERK and p38 MAPKs stimulates BK channel activity, indicating that these kinases inhibit the channel in both PCs and ICs (Li et al., 2006). They also showed that a high K^+^ diet decreases the phosphorylation of ERK and p38 MAPKs (Li et al., 2006).

pH modulates BK channel activity by strongly and reversibly inhibiting the channel when extracellular pH falls below 5, affecting its voltage sensor domain. Extracellular H^+^ promotes the closed conformation of the channel and directly inhibits its pore (Zhou et al., 2018). Increased intracellular H^+^ reduces BK channel current, while higher intracellular K^+^ levels relieve this inhibition, suggesting a competitive inhibition with K^+^ by H^+^ (Brelidze & Magleby, 2004).

## 4 FIKS IN THE DISTAL NEPHRON

Micropuncture studies (Khuri et al., 1975) and in vitro microperfusion studies (Engbretson & Stoner, 1987) dating back to the mid‐1970s demonstrated that the rate of active K^+^ secretion was directly influenced by the rate of tubular fluid flow and Na^+^ delivery. ENaC itself is a mechanosensitive channel responsive to flow‐induced shear stress, but not in Ca^2+^‐dependent manner (Carattino et al., 2004; Kashlan et al., 2024; Morimoto et al., 2006). An increase in distal fluid delivery not only provides more Na^+^ as a “substrate” for ENaC, but increases the channel *P*_*O*_. This enhances the lumen‐negative potential and depolarizes the PC apical membrane, promoting K^+^ efflux through ROMK channels. ROMK is abundantly expressed in PCs and displays a constitutively high *P*_*O*_ when assessed via patch clamp (Frindt & Palmer, 1989; Nesterov et al., 2022; Wang et al., 1990). While ROMK‐mediated K^+^ secretion in PCs had long been considered to be the primary K^+^ secretory pathway in the ASDN, its role was challenged over the decades by a number of observations, including the following. Amiloride‐insensitive K^+^ excretion has been demonstrated in dietary K^+^‐loaded mice, suggesting that at least some portion of the renal K^+^ secretory capacity is ENaC‐independent (Yang et al., 2020). Although FIKS in the rabbit CCD is inhibited by ENaC blockade in microperfusion studies (Liu et al., 2007), the kidneys can also readily adapt to prolonged dietary Na^+^ restriction associated with K^+^ loading, as is reflected in the diet of the Yanomami people of South America (Cornelius et al., 2016; Mancilha‐Carvalho Jde, 2003). Sustainability of this diet necessitates that K^+^ excretion be at least partially uncoupled from Na^+^ reabsorption. Furthermore, the classical ENaC/ROMK coupling mechanism is not by itself sufficient to explain why Type II Bartter's syndrome (ROMK deficiency) leads to *hypo*‐ rather than *hyper*kalemia during later stages of development and into adulthood (Bailey et al., 2006).

## 5 ROLE OF BK CHANNELS

Direct evidence for apical BK channel involvement in FIKS was found in early studies using global BKα^−/−^ mice, which were shown to have an attenuated kaliuretic response to vasopressin receptor antagonism (Rieg et al., 2007), a maneuver that triggers increased fluid flow rates. However, interpretation of these findings is complicated by the widespread expression of BK channels throughout the body, including in the renal vasculature and the aldosterone‐producing *zona glomerulosa* cells of the adrenal cortex (Sausbier et al., 2005; Vassilev et al., 1992). Indeed, these global BKα^−/−^ mice exhibit primary hyperaldosteronism. Off target effects on aldosterone signaling or renal hemodynamics are major confounding factors associated with a global loss of BKα expression. More recently, isolated CCDs from IC‐BK⍺^−/−^ mice were shown to exhibit a complete loss of FIKS, measured by ex vivo microperfusion, in response to an increase in luminal flow rate (Carrisoza‐Gaytan et al., 2020). Charybdotoxin‐sensitive whole‐cell currents were also abolished specifically in ICs in CCDs from these animals, indicating that BK channels in ICs mediate FIKS. Importantly, flow‐induced Ca^2+^ signaling was still evident in both ICs and PCs in CCDs from these mice, suggesting that attenuated FIKS was due to a loss of the apical K^+^ channel conductance and not an impairment of mechanosensation. IC‐BKα^−/−^ mice likely compensated for this deficit through other renal K^+^ handling pathways, as urinary K^+^, Na^+^ and overall volume output were not altered at baseline or when maintained on a high K^+^ (5% KCl) diet. Male IC‐BKα^−/−^ mice were slightly hyperkalemic following K^+^ loading, although the same was not observed in females. A limitation of this model, particularly regarding whole animal studies, is that expression of the Cre recombinase used to knock out BK⍺ was driven by the vacuolar H^+^‐ATPase B1 subunit promoter. Using a reporter mouse line, we noted that the Cre recombinase was primarily expressed in ICs (Carrisoza‐Gaytan et al., 2020), although others have reported Cre recombinase expression in 50% of PCs within the CNT (Miller et al., 2009).

ICs consist of acid‐secreting type A ICs, base‐secreting type B ICs, as well as a population of nonA, nonB ICs. We are not aware of evidence suggesting that BK‐dependent K^+^ secretion is restricted to a specific IC subtype. While PCs express basolateral membrane Na^+^,K^+^‐ATPase, providing a mechanism for the active transport of K^+^ into PCs to maintain a high intracellular [K^+^], there is a paucity of basolateral Na^+^,K^+^‐ATPase expression in ICs (Palmer & Frindt, 2007). A basolateral Na^+^,K^+^,2Cl^−^ co‐transporter (NKCC1) provides a pathway for K^+^ entry into ICs and is required for FIKS (Liu et al., 2011).

One puzzling finding from these experiments is that the whole cell currents measured from ICs were sensitive to 100 nM charybdotoxin, despite evidence that BKα colocalizes only with BKβ4 subunits in this cell type (Grimm et al., 2007; Wen et al., 2013). Cumulative evidence suggests that inhibition of BKα/β4 channels requires >1 μM concentrations of charybdotoxin or iberiotoxin (Lippiat et al., 2003; Wang et al., 2014). One possibility is that BKβ4 subunits do not undergo typical glycosylation in ICs, leaving the channel's sensitivity to peptide toxins intact. Another possibility is that BKβ4 does not associate with BKα subunits in these cells, but rather fulfills some other function independent of BK channel activity. However, BKβ4^−/−^ mice display an impaired adaptation to dietary K^+^ loading, arguing against this as an explanation. Alternatively, if BKγ subunits are also expressed in ICs, it is possible that co‐assembly of BKα with both β4 and γ subunits may confer sensitivity to peptide toxins. However, the precise combination of BK channel subunits that assemble in ICs remains unknown.

## 6 ROLE OF Ca^2+^ SIGNALING

A rapid increase in tubular flow rate subjects both PCs and ICs to three types of mechanical forces: (i) fluid shear stress, (ii) circumferential stretch, and (iii) shear or drag forces on apical cilia of PCs and microvilli/microplicae of ICs (Liu et al., 2003). These hydrodynamic forces trigger biphasic increases in [Ca^2+^]_i_, essential for BK channel‐mediated FIKS in ICs (Liu et al., 2007). The BKα‐subunit possesses high‐affinity Ca^2+^‐binding sites and is generally activated by an increase in [Ca^2+^]_i_. Sensitivity to Ca^2+^ is modulated by association with the different subunits. For instance, the β1 subunit increases its Ca^2+^ affinity (Cox & Aldrich, 2000), whereas the β4 subunit either positively or negatively changes the channel's sensitivity, depending on the [Ca^2+^]_i_ concentration (Brenner et al., 2000). It also has been shown that association with the Ɣ3 subunit decreased BK channel sensitivity to Ca^2+^ (Li & Yan, 2016).

**FIGURE 2:**
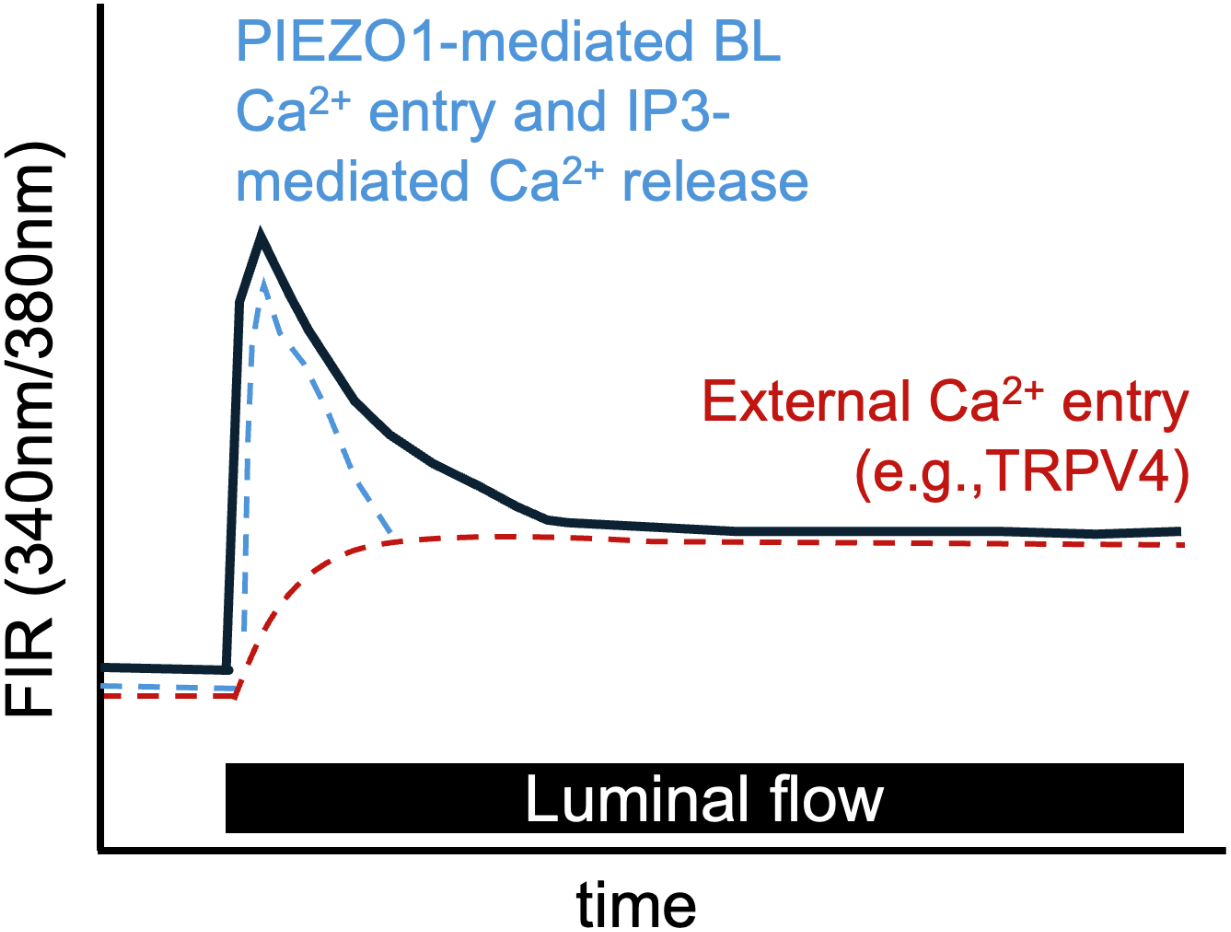
Effect of an increase in luminal flow rate on [Ca^2+^]_i_, in the isolated microperfused CCD. Representative tracing of fura‐2 fluorescence intensity ratios (FIRs), equivalent to [Ca^2+^]_i_, showing the biphasic increase in [Ca^2+^]_i_ in ICs in response to an increase in luminal flow rate (solid black line) (Carrisoza‐Gaytan et al., 2024). The initial peak in [Ca^2+^]_i_ is triggered by opening of basolateral PIEZO1 channels, allowing basolateral Ca^2+^ entry, and IP3‐sensitive internal store release (blue dashed line) (Carrisoza‐Gaytan et al., 2024; Liu et al., 2003). This is followed by a rapid decay to a sustained plateau elevation throughout the period of high flow, reflecting external Ca^2+^ entry, mediated in part by TRPV4 (red dashed line) (Taniguchi et al., 2007).

The biphasic flow‐induced Ca^2+^ response in distal tubular epithelial cells consists of an initial peak in [Ca^2+^]_i_, reflecting extracellular Ca^2+^ entry at the basolateral membrane coupled with release of IP_3_‐sensitive internal Ca^2+^ stores (Liu et al., 2003). Following this peak, there is a rapid decay to a sustained plateau throughout the period of high flow, reflecting luminal Ca^2+^ entry in part mediated by the mechanosensitive TRPV4 channel (Figure 2) (Taniguchi et al., 2007). Microperfusion studies, performed on microdissected CCDs, showed that addition of the TRPV4 agonist 4αPDD to the lumen enhanced FIKS. In contrast, CCDs from global TRPV4^−/−^ mice model did not show an increase in net K^+^ secretion in response to flow, underscoring the critical role of TRPV4 in FIKS (Taniguchi et al., 2007). Later studies in tubular specific knockout mice (TRPV4^fl/fl^‐Pax8Cre) confirmed that urinary K^+^ excretion in response to K^+^ loading was lower in the TRPV4^fl/fl^‐Pax8Cre mice, concomitant with elevated plasma K^+^ levels (Stavniichuk et al., 2023).

A recent study demonstrated that another mechanosensitive channel, PIEZO1, is expressed in both ICs and PCs (Dalghi et al., 2019). PIEZO1 acts as a key mechanosensor in ICs, facilitating flow‐induced basolateral Ca^2+^ entry, triggering an increase in [Ca^2+^]_i_ and BK channel‐mediated FIKS (Carrisoza‐Gaytan et al., 2024). In that same study, activation of PIEZO1 by an increase of luminal flow or basolateral addition of the channel agonist Yoda1 led to equivalent increases in [Ca^2+^]_i_ in both ICs and PCs (Carrisoza‐Gaytan et al., 2024). Targeted genetic deletion of *PIEZO1* in ICs resulted in a loss of the flow dependent increase in [Ca^2+^]_i_ in ICs and of FIKS in microperfused CCDs, highlighting PIEZO1's essential role in FIKS (Carrisoza‐Gaytan et al., 2024). However, a different study showed that agonist‐induced PIEZO1‐mediated Ca^2+^ influx is significantly greater in PCs than in ICs and is differentially regulated by changes in fluid flow, with a significant PIEZO1 activation observed in PCs but not in ICs (Pyrshev et al., 2024). These contrasting observations may be attributed to differing methodologies: the former study assessed the impact of an increase in luminal flow in isolated microperfused CCDs obtained from wild type and IC PIEZO1 knockout mice, while the second involved a PIEZO1 activator, as well as pre‐injecting mice with the diuretic furosemide or treating them with a high K^+^ diet to increase flow in the distal nephron (Carrisoza‐Gaytan et al., 2024; Pyrshev et al., 2024). A high K^+^ diet enhances distal tubular flow in vivo via inhibition of salt and water reabsorption in the proximal tubule (Brandis et al., 1972) and loop of Henle (Battilana et al., 1978; Brandis et al., 1972; Stokes, 1982; Unwin et al., 1994). Pyrshev et al. also underscored the link between PIEZO1 and TRPV4, demonstrating that TRPV4 activation is facilitated by initial transient PIEZO1 activation (Pyrshev et al., 2024). PC‐specific *PIEZO1* knockout mice have not yet been generated but will provide important new insight into specific roles of PIEZO1 in PCs.

Although it is now evident that BK channels in ICs are responsible for FIKS, their role in PCs remains uncertain. In PCs of rabbits fed a standard K^+^ diet, immunodetectable BKα, and conducting BK channels are concentrated within the apical cilia, and chemical deciliation of PCs did not impair FIKS, consistent with the role of ICs BK channels in mediating FIKS (Carrisoza‐Gaytan et al., 2017). However, chemical deciliation of PCs led to the loss of the initial high‐amplitude [Ca^2+^]_i_ peak of the flow‐induced [Ca^2+^]_i_ response in both ICs and PCs (Carrisoza‐Gaytan et al., 2017). Further studies are required to determine the role of BK channel in PCs.

## 7 REGULATION BY DIETARY K^+^

Animals fed a K^+^‐rich diet maintain K^+^ balance in part via BK channel‐mediated secretion in the ASDN (Cornelius et al., 2012; Li et al., 2006; Welling, 2016). This supplements ROMK activity in PCs, which is also upregulated via aldosterone signaling during dietary K^+^ loading (Welling, 2016). However, a direct link between aldosterone and BK channel activity in ICs has not been established. There have been conflicting reports concerning the relative importance of dietary K^+^ per se, versus the hyperaldosteronemic response induced by high K^+^ feeding. Evidence against aldosterone, but rather blood K^+^ itself, as the primary regulator of BK channel‐mediated K^+^ secretion can be inferred from the observation that rabbits fed a low Na^+^ diet designed not to alter blood [K^+^] did not exhibit iberiotoxin‐sensitive K^+^ secretion in microperfused tubules, despite having elevated aldosterone (Estilo et al., 2008). However, others have shown increased FIKS in microperfused CCDs isolated from Na^+^‐depleted K^+^‐supplemented (hyperaldosteronemic) rabbits compared to controls (Engbretson & Stoner, 1987). We recently showed that mice fed a Na^+^‐deficient diet also exhibit robust FIKS (Ray et al., 2024). Aldosterone signaling has been shown to regulate BKα subunit expression and K^+^ secretion in mice, and the effects of K^+^ loading are attenuated by spironolactone (Wen et al., 2013).

ICs exhibit little active 11β‐hydroxysteroid dehydrogenase (11β‐HSD) expression (Kyossev et al., 1996; Naray‐Fejes‐Toth et al., 1994), which inactivates glucocorticoids and enables sensitivity of the mineralocorticoid receptor to aldosterone. However, single cell RNAseq studies have found 11β‐HSD gene expression in ICs (Chen et al., 2017; Stewart et al., 2019). It is possible that mineralocorticoid receptor (MR) signaling in ICs is driven by glucocorticoids, as is the case in the upstream DCT2/CNT segments. However, glucocorticoid‐driven MR activation should not vary in response to dietary salt intake. This provides additional support in favor of an aldosterone‐independent mechanism of regulation. Interestingly, MR expressed specifically in ICs can undergo phosphorylation at S843, which disrupts ligand binding and prevents nuclear translocation (Shibata et al., 2013). This mechanism was recently shown to be Unc‐51‐like kinase (ULK1)‐dependent (Shibata et al., 2018) and regulated, at least in part, by metabolic target of rapamycin (mTORC2) signaling (Ali et al., 2024). mTORC2 signaling may, in turn, be regulated directly or indirectly by blood K^+^ levels or aldosterone. Future studies will uncover the details surrounding this complicated regulatory cascade.

Similar to a chronic feeding of a high K^+^ diet, a low K^+^ diet induces a significant elevation of tubular flow in the distal nephron (Rieg et al., 2007; Stavniichuk et al., 2023) but does not induce FIKS as urinary K^+^ excretion is considerably decreased to maintain a plasma [K^+^] levels within a physiological range (Giebisch, 1998). A low K^+^ diet also induces, through a GDF15‐dependent mechanism (Lasaad et al., 2023; Lasaad & Crambert, 2024), an increase in number of a subpopulation of ICs, those cells responsible for FIKS. This raises the following question: What could protect against excessive K^+^ secretion in this context; where tubular flow and ICs number are increased, but K^+^ needs be retained? First, a low K^+^ diet has been shown to decrease expression of BK⍺/β2‐4 mRNA levels in rabbits CCDs (Najjar et al., 2005). Additionally, BK⍺ protein is minimally expressed at the apical membrane of ICs of low‐K^+^ diet fed‐rabbit CCDs (Carrisoza‐Gaytan et al., 2017). Interestingly, Elabida et al. showed that a low K^+^ diet increases progesterone levels in male mice (Elabida et al., 2011) that could result in a decreased K^+^ conductance as observed in MDCK cells (Steidl et al., 1991). Progesterone has also been shown to bind to BK channels and inhibit channel activity when expressed in X. *laevis* oocytes (Wong et al., 2008). It should be noted that a different study showed that supraphysiological levels of progesterone activate mouse cerebrovascular myocyte BK channels through the β1 subunit which, in the distal nephron, is expressed in PCs (Carrisoza‐Gaytan et al., 2017; North et al., 2023). Moreover, progesterone inhibits TRPV4 in different tissues (Jung et al., 2009). If a similar mechanism exists in the ASDN in response to a low K^+^ diet, this would lead to a reduced sensitivity to tubular flow (and thus reduced FIKS) but may also contribute to the activation of K^+^ reabsorption. Indeed, tubular deletion of TRPV4 increases the expression of the H^+^,K^+^‐ATPase type 2 (Tomilin et al., 2019), which contributes to K^+^ reabsorption in ICs (Lasaad et al., 2023). Based on these studies, we can hypothesize a potential inhibitory role of TRPV4 on the H^+^,K^+^‐ATPase, which may be released by a progesterone‐dependent decrease of TRPV4 expression during a low‐K^+^ diet. Further studies are needed to assess TRPV4 expression and its potential regulation by progesterone within the kidney during a low K^+^ diet, and to determine the molecular mechanisms by which TRPV4 deletion increases the H^+^,K^+^‐ATPase activity.

In summary, it is now well‐established that BK channels play a crucial role in K^+^ homeostasis in the kidney. Recent studies have revealed the pivotal role of BK channels, specifically in ICs, in mediating FIKS through a Ca^2+^‐dependent mechanism. Additionally, PIEZO1 has been identified as a key mechanosensor driving the initial Ca^2+^ influx required for FIKS, with TRPV4 working downstream of PIEZO1 to contribute to Ca^2+^ influx as well. However, many questions remain. Is FIKS dependent on the activity of a particular BKα splice variant (e.g., STREX/ZERO/e23)? Do PCs and ICs express similar or distinct BKα splice variant populations? What mechanistic and possibly cell‐specific roles do the auxiliary β  or γ subunits fulfill? Does FIKS occur in the DCT2/CNT segments where ENaC activity is driven by glucocorticoids, rather than by aldosterone? If so, do glucocorticoids directly regulate FIKS? What role, if any, do PC‐localized BK channels play in the renal regulation of ion transport? These questions will surely be addressed in the coming years, as we continue to advance our understanding of molecular mechanism underlying FIKS, and the role(s) that BK channels in the ASDN play in health and disease.

## CONFLICT OF INTEREST STATEMENT

The authors have no conflicts of interest.
